# Identification of heart rate change during the teaching process

**DOI:** 10.1038/s41598-023-43763-x

**Published:** 2023-10-04

**Authors:** Jan Francisti, Zoltán Balogh, Jaroslav Reichel, Ľubomír Benko, Kristián Fodor, Milan Turčáni

**Affiliations:** 1 Department of Informatics, Faculty of Natural Sciences and Informatics, Constantine the Philosopher University in Nitra, Nitra, Slovakia; 2 Kandó Kálmán Faculty of Electrical Engineering, Óbuda University, Budapest, Hungary

**Keywords:** Computer science, Information technology, Physiology, Psychology

## Abstract

Internet of Things (IoT) technology can be used in many areas of everyday life. The objective of this paper is to obtain physiological functions in a non-invasive manner using commonly available IoT devices. The aim of the research is to point out the possibility of using physiological functions as an identifier of changes in students’ level of arousal during the teaching process. The motivation of the work is to find a correlation between the change in heart rate, the student’s level of arousal and the student’s partial and final learning results. The research was focused on the collection of physiological data, namely heart rate and the evaluation of these data in the context of identification of arousal during individual teaching activities of the teaching process. The experiment was carried out during the COVID-19 pandemic via distance learning. During the teaching process, individual activities were recorded in time and HR was assigned to them. The benefit of the research is the proposed methodology of the system, which can identify changes in students’ arousal in order to increase the efficiency of the teaching process. Based on the results of the designed system, they could also alert teachers who should be able to modify their teaching style in specific situations so that it is suitable for students and provides a basis for better teaching and understanding of educational materials. The presented methodology will be able to guarantee an increase in the success of the teaching process itself in terms of students’ understanding of the teaching materials.

## Introduction

Elements of the Internet of Things (IoT), including sensory networks, are all around us. There are more and more smart devices every day that could be included in the Internet of Things technology, whether they are smart devices in households or the workplace. The mentioned technology does not bypass education, even teaching processes.

Sensory networks can be considered as a tool that enables information exchange between devices that can communicate. The combination of the mentioned devices can create a complex system. The use of sensory networks in the learning process can be a useful tool to optimize the teaching process itself and to support education.

The aim of involving sensory networks in the teaching process is to optimize the teaching process itself for the teacher. Sensory networks in the teaching process can also provide information to the teacher about the student’s feelings. Our effort in the article will be to provide the teacher with information about the change in the degree of arousal of students with the help of sensory networks.

The teaching (educational) process is gradually beginning to focus on the personality of the student, where the teacher acts as a tutor. E-learning has become part of today’s education also thanks to its diverse use, from the presentation of digital content to teaching management systems (LMS)^1^. The current situation with the COVID-19 pandemic forced most school facilities to switch to distance education.

Emotions are important in the student’s learning and significantly affect the results of their learning activities. For example, positive emotions (joy, interest, inspiration) improve the procedural side^2^. Emotions are extremely important for the teaching process and students learning and therefore require special attention. Good motivation promotes emotionality and emotional activity which is the basis of good results^3,4^.

During the teaching process, situations arise in which students are aroused, e.g. exam tests, answering questions and more. That is why it was our effort to reveal situations in which students show a higher degree of arousal, to adjust the teaching process in such a way that students do not show higher levels of stress.

Based on the findings of research conducted by other authors, described in the “Related work” section, we learned that physiological functions can be used as an identifier of changes in the degree of arousal. In our research, we used the physiological function of the heart rate (HR). To be able to measure the HR of students and not disturb them in class, we used wireless smart wristbands which contained a sensor capable of measuring the mentioned physiological function^5^.

The research was carried out in detail during the subject Operating Systems which was completed by students of the second year of bachelor’s studies in the field of Applied Informatics. The research was carried out throughout the term. The course was divided into two parts, lectures and exercises, during which students performed various activities. As the research was conducted during the COVID-19 pandemic, the teaching process took place in a distance form. At the beginning of the research, students were given detailed instructions. The research involved 24 students. Students wore a wristband throughout the teaching process, and all data obtained was recorded via a mobile device and then sent for processing to the cloud storage. All personal data has been processed in accordance with the GDPR Regulation. Each participant was informed about the acquisition of physiological data (HR) and all participants’ data were anonymized.

The obtained data from the wristbands were subsequently evaluated and described in detail in the “Results” section. The mentioned section describes the implemented activities during the teaching process and the results from the point of view of student arousal. Other obtained research results are also described.

The main motivation for the research was the possibility of identifying changes in the degree of arousal in students during the teaching process using smart wristbands, the physiological function of heart rate as identifiers, the student’s level of arousal and the student’s partial and final learning outcomes.

The article aims to use and apply sensory networks in the teaching process to bring teachers recommendations on how to optimize the teaching process itself for the benefit of students. The aim of the research was also to verify whether it is possible to use separate information about the student’s HR obtained from a smart wristband in real-time to distinguish students’ degree of arousal and find associations between these changes in degrees of arousal and different types of activities during the teaching process.

As part of the research, we used 20 bracelets that recorded heart rate and presented individual sensors. The bracelets used in the research were chosen based on the conducted research^6^. Subsequently, the bracelets sent data to the mobile device for analysis. The mentioned devices together formed a sensory network.

The research was carried out during the COVID-19 pandemic, when the lockdown was mandatory and based on the valid schedule, distance learning was carried out. During the teaching process, the teacher was present on the set date and the teaching consisted of several activities that are mentioned in the “Materials and methods” section. During the teaching process, the selected students had their heart rate measured as a physiological function based on bracelets. Since the individual activities were recorded in time during the teaching process, it was possible to know what the students were doing at all times and how the given activity was reflected in their heart rates. Samples of individual records are presented in tables and visualized in the respective figures.

## Related work

### Sensory networks

Sensory networks can be used in education and the teaching process. Sensory networks in the teaching process aim to provide information to the teacher about the feelings of students. Our effort in the article will be to provide the teacher with information about changes in the degree of arousal of students with the help of sensory networks. Technologies provide a guarantee of creating an environment that supports the acquisition of knowledge in a new and effective way consistent with students’ expectations^7,8^.

As part of the Smart Campus project^9–11^, teaching took place through an e-learning course where students could connect from any place with an internet connection. In this way, students could be part of the curriculum and communicate with the teacher outside the classroom.

### Internet of Things as teaching process tool

The Internet of Things is now becoming an integral part of everyday life. Taking advantage of IoT opens the door to a new world where many things can be handled much more efficiently and easily^12–15^.

Elements of the IoT in the teaching process can be used as a teaching process tool. These are devices that support IoT technology, such as smart devices in the form of wristbands and other items that students can wear during the teaching process^16–18^.

IoT devices are not directly connected to the Internet. A Bluetooth connection is used to transfer information with a control device, computer or smartphone that is connected to the Internet and can move data further, either to local storage or to the Cloud^19–21^.

### Emotions and the importance of their evaluation in the context of education

Emotions are the basis of human experience, although they are difficult to define, recognize and classify. Feelings and emotions play an important role in human life because they are part of the motivational structure. Emotions have a significant impact on the cognitive processes of the human brain, such as learning, memory, perception and problem solving^22^.

Feelings have different internal and external manifestations. People can see different changes when they experience some emotions, for example when they are happy, they have a smile on their face^23–27^.

The question of evaluating human emotions is a very interesting topic that has been gaining more and more attention in recent years. It is necessary to mention the specific connection of several unrelated areas (automation) and areas such as computer science and psychology. Therefore, it has major potential to improve human–computer interaction in various areas, including the learning environment^28,29^.

During the teaching process, students also manifest different emotional states in different situations. Therefore, it is important to explore ways to help identify emerging emotional states to ensure that students have positive feelings during class to get the most out of the teaching process^30–32^.

Emotions and motivation are important prerequisites, mediators and outcomes of learning and achievement^33^. Based on the authors’ research, we found that emotions affect people’s cognitive abilities. The authors^34,35^ conducted an experiment on medical students and pointed out that emotions can interfere with cognitive processes such as perception or attention due to studying online materials about a patient’s problem. If attention is diverted to other activities, such as processing emotions or integrating information, attention itself is suppressed. Cognitive abilities are a direct predictor of study results^36^.

Authors^37^ involved medical students in their experiment. The research results showed that students reported a significantly higher habitual level of reappraisal than suppression of emotion regulation strategies. A higher level of habitual reappraisal significantly and positively predicted students’ self-evaluation. Conversely, higher levels of habitual suppression significantly and positively predicted students’ self-reported anxiety, shame, and hopelessness.

In the research, the authors^38^ pointed out that soft skills were directly positively related to students’ emotions related to achievement, learning, motivation and life satisfaction and were indirectly related to academic learning and motivation outcomes.

### Measurement and detection of emotional changes

The ability to recognize emotions is one of the characteristics of emotional intelligence. In particular, physiological signals are widely used to detect and classify affective conditions^22,39^. Nowadays, with rapid advances in intelligent technology, computer-aided recognition of emotions using physiological signals is attracting growing academic interest^40^.

The measurement of stress can be performed according to three main methods, including psychological, physiological and behavioral. According to the authors’ experiments^41–44^, the most accurately developed systems for detecting stress are through physiological signals. The results show that the ECG (Electrocardiogram), especially HRV (Heart Rate Variants) and EDA (Electrodermal Activity) functions, are the most accurate signals of recognizing physiological stress.

According to authors Bustos et al.^45^, ECG (Electrocardiogram) signals such as heart rate or heart rate variability are associated with stress, surprise, relaxation and concentration. Meanwhile, PPG (Photoplethysmography) signals such as blood volume or oxygen saturation are related to stress, laughter, interest and frustration. In both cases, physiological signals make it possible to determine the involvement of pupils in educational activities.

Based on research conducted by other authors, we can state that physiological functions can be used as a tool to identify changes in emotional states, in our experiment specifically changes in degrees of arousal^46–51^.

Both students and teachers experience different kinds of emotions, from positive ones like joy to negative ones like anger or anxiety. Although emotions are ubiquitous, they have been relatively neglected in educational research. Emotions highlight students’ attention and initiate the learning process. They influence what we learn and remember. According to the conducted studies, it was confirmed that emotions have a significant role in the teaching process^52–55^.

In addition to their role in the teaching process, emotions are also a fundamental component of effective teaching. Research indicates that teachers’ emotions have a key influence on the entire teaching process^56,57^. The way teachers experience and express emotions directly affects their pedagogical approach, which has consequences not only for students’ behavior, but also for the transfer of emotions between students themselves^58,59^.

### Use of intelligent equipment for measurement of physiological functions

There are many types of devices that have similar functions that are commonly used. These are devices capable of measuring the required physiological functions and displaying them, or sending them to a central device, e.g., a computer for further processing of measured data. A large amount of scientific literature contains descriptions or designs of sensors and biosensors for measuring physiological functions^60–64^. Specifically, we will deal with devices that support these features. These are devices that take the form of wristbands or other wearable devices that we can wear for a long time, with the idea that we can perform other work and at the same time the device will measure functions from the body. These devices are characterized by interoperability, which ensures that they can cooperate and communicate with devices of another type and are connected by a single central computer. For example, one device measures HR, the other body temperature, and a central computer can process various data and create correlations^65–67^.

Authors^47,68–70^ used wristbands to measure HR during sports. Athletes wore wristbands and performed assigned tasks, such as running or others. As the first sensor, it began measuring heart activity and moving the data further. The chip processed the data and kept it in memory. After connecting the wristband to the central computer, the data was sent for processing. The system server received the data from the wristband and processed it into a defined form. It also allows the terminal to access the processed data.

Stress can also be measured using intelligent wearable devices^5,71^. In the research, the authors used wearable devices that can measure heart activity (HR, HRV) and identify the presence of stress based on the obtained values.

Author^72^ explains that the rapid development of wristbands has made it possible to continuously record neurophysiological signals in the natural environment, for example in a classroom. The study conducted by Zhang aimed to examine the neurophysiological correlates of the academic results of high school students. The HR was used as an identifier in the research and the result of the research revealed significant correlations between academic performance, which is reflected in the score of students’ final exams and physiological function.

The results of Zhang’s study are also confirmed by the author^73^ who states that the latest advances in sensor technology make it possible to examine students’ emotional and cognitive states.

Based on the research performed by the above-mentioned authors, we found that sensory networks are a suitable tool for identifying and analyzing changes in emotional states, which can be used in the teaching process. Processed information obtained from individual sensors can be provided to teachers through graphical visualization, or specific recommendations. Based on the obtained results, the teacher can adjust the teaching process to ensure maximum effectiveness of teaching. Studies also show that acquired physiological functions through sensors can be transformed into changes in the emotional state of students, which are an indicator of how students perceive the teaching process. Based on the analysis of the current state, we found that by measuring the HR, it is possible to identify changes in the degree of arousal of students during the teaching process.

## Materials and methods

**Figure 1:**
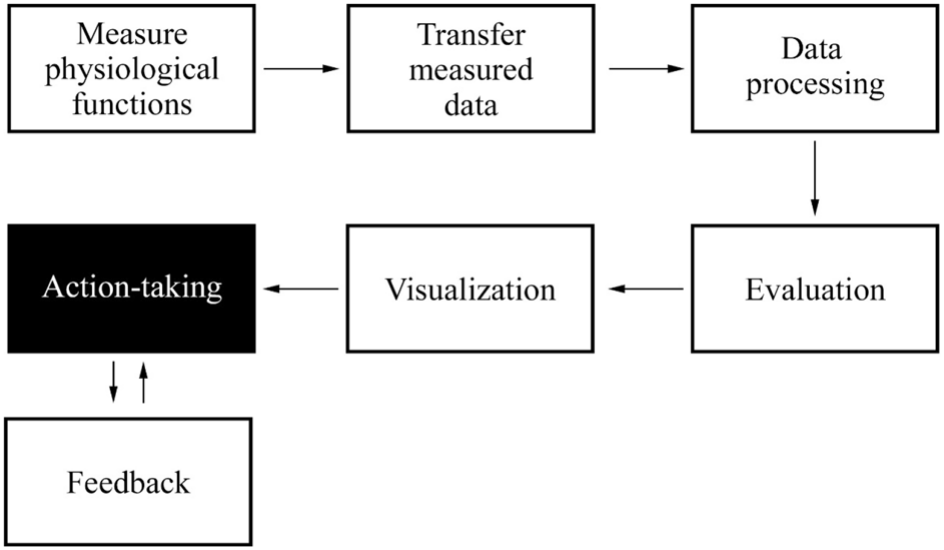
Conceptual model of a complex system. The comprehensive system contains a sequence of steps by which the research was conducted.

We can classify this research as biomedical research which was carried out with the approval of the ethics committee of the Constantine the Philosopher University in Nitra, which ensures that the rights of human subjects (volunteers) were respected in the studies. This consent has been granted under the number UKF-2022/954-2:191013 and informed consent was obtained from all participants. All methods were carried out in accordance with relevant guidelines and regulations. The condition for achieving the results of our research is the process of procedures shown in Fig. 1.

Based on the studied scientific articles focused on the teaching process using sensory networks, it was found that there is a lack of research focused on the teaching process, which can be monitored based on sensory networks using smart bracelets as sensors for collecting the physiological function of the heart rate. Similar to the research from the authors^74^, it was on the basis of the findings that a research gap was established, which we addressed.

To the best of our knowledge, there are only a limited number of literature sources such as the research by Carroll et al.^75^ or Giannakos et al.^76^ which deal precisely with the use of sensory networks in combination with the teaching process, where smart bracelets were used to measure the physiological function of the heartbeat. In this part, we are dealing with the creation of a system that would be able to ensure the measurement of physiological function, the analysis of the obtained data and help the teachers in the teaching process.

The main goal of the research was to measure HR in students during the teaching process, where each activity was time-matched with HR data. Based on the obtained data, the effort was to identify changes in HR values and to observe the cause of HR value changes in the teaching process. The benefit of the research is a system that is able to identify changes in the emotional state of students in order to increase the effectiveness of the teaching process.

To implement the proposed system, it was first necessary to take a few steps. The research methodology was divided into several parts:

- identification of change in the degree of arousal—since the research was aimed at identifying changes in the degree of arousal, we focused on research with the same issues,
- selection of physiological functions—based on the studied literature and research, we found that physiological functions are a capable tool to identify changes in the degree of arousal, and therefore we have used it in our research,
- type of physiological functions—from the research by other authors, we found that HR is a suitable type of physiological function,
- method of measuring physiological function—for measuring the physiological function of the HR, we chose an intelligent wristband containing the corresponding sensor,
- choosing the right type of wristband—based on the research, we chose a smart wristband with the most accurate sensor for the research^6^,
- selection of the subject of research—since we researched the teaching process, we chose the e-course in Moodle (Virtual Learning Environment—VLE) at our university from the subject Operating Systems (https://edu.ukf.sk/course/view.php?id=1438), belonging to the group of compulsory subjects,
- creation of activities of the subject—based on the syllabus of the subject, through which we carried out the research, certain activities were created,
- division of students into groups—students were divided into two groups, where one group represented an experimental group and the other a control group,
- data cleaning and preparation—all obtained data during the whole teaching process from the wristbands and the course were cleaned and prepared for analysis,
- data analysis—after data cleaning, analyses were performed, where we compared students as a whole and their behavior in the teaching process, we also compared two groups of students (experimental and control group),
- evaluation of results—through the performed analyses we evaluated and interpreted the obtained results,
- recommendations for optimizing the teaching process—the research and the obtained results were a key part of the creation of recommendations on how to optimize the teaching process to achieve higher quality in terms of obtaining higher student evaluation.
- Based on the developed methodology, all parts were elaborated in detail in the later sections of the article.

### Transmission of measured data

The wristbands selected in the research did not have a built-in memory, the measured data were stored directly in the application on a mobile device. Bluetooth technology was used to transmit the acquired data which ensured the wireless connection of the smart wristband with the mobile device.

The MiTools application was used for the wristbands manufactured by Xiaomi. The continuous HR monitoring function was used in the research. If HR does not change, the same value will not be sent to the application. The application also has a function for sending the obtained data, which was used in the research.

### E-learning course operating systems in virtual learning environment

The aim of the course called operating systems (OS) is to obtain basic information about the construction of operating systems and the theoretical foundations of computer science which are necessary for understanding the construction and functionality of current operating systems.

Each of the topics contained a theoretical part, a presentation from the lecture and a book activity that contained the teaching materials covered in the lecture.

The course included various activities in the form of practical tasks that were carried out during the exercises and the Assignment activity where students recorded tasks for which they earned points. At the beginning of each exercise, students performed a self-test which consisted of materials covered in the last lecture.

The self-test activity (in the VLE Moodle Quiz module) was created from the questions contained in the lectures. Each test contained 10 questions and was set to 7 min. A time interval of 7 min was created based on the testing self-test, which was used to calculate the average time required to perform the self-test. One self-test was created for each week. During the test, the students’ HR was measured. The data obtained were tabulated and the average beats per minute was used for analysis. The test was set up sequentially and students were not allowed to return to the previous question, which they were notified of before each self-test^77^.

All activities performed during the exercise were recorded in the table together with time to synchronize with other obtained data (data from the log file of the e-learning course and data obtained from smart wristbands). A scoring system was created in the course, according to which students gained points during the whole semester.

Students were divided into two groups, of which one group was experimental and the other control. The experimental group differed from the control group by a scoring system during the semester. At the beginning of the exercise, the control group carried out a self-test as a repetition of the teaching materials taken over at the last lecture and the points obtained were not included in the final grade. The experimental group performed self-tests as a part of the subject and according to the scoring system, the points obtained from the self-tests were counted in the final grade. In this way, we wanted to indirectly evoke in the experimental group the motive of trying to get the highest number of points from the self-tests. We also observed changes in the level of arousal during the writing of self-tests for both groups.

### Cleaning and preparation of data

**Figure 2:**
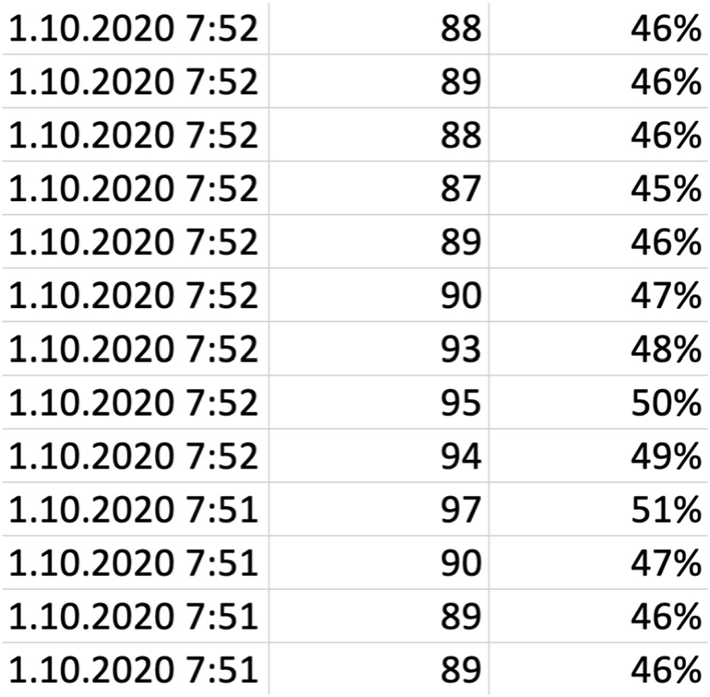
Part of the export data from a wristband. The content of the image represents the date, the heart rate value, and the percentage evaluation of the heart rate elevation.

We exported data from each wristband in the manner described in more detail in the previous subsection B. We obtained information about each student’s HR. The data from each wristband was in a standard csv format and an example is depicted on Fig. 2. Another source of data was timestamps of individual activities, which were performed during the semester in classes, whether exercises or lectures. They were also a source of data on the self-test activity that the students completed during the exercise. From the VLE Moodle, we exported information on the time intervals in which students completed the self-tests. These multiple sources had to be combined into a single file for further analysis. In the process of data preparation, we were inspired by the method of data preparation from log files originating from web servers^78–83^.

**Figure 3:**
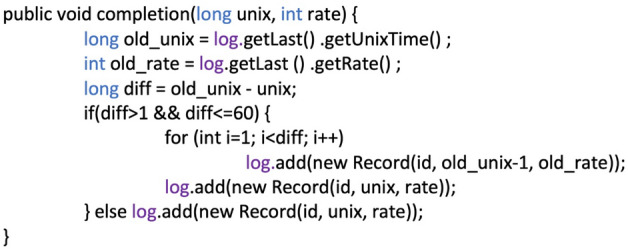
Pseudocode for filling in missing records from wristband log files.

We proceeded with the following steps when pre-processing the input files:

1. *Data acquisition* Includes the export of data from smart wristbands (Fig. 2) and VLE, the export of timestamps of activities during exercises, and lectures that represent our input data. The input data contained a total of approximately 250,000 records that were obtained from wristbands.
2. *Record cleaning* Smart wristband records also included records from non-teaching times, and these records were not relevant to our research. For this reason, the timing of the individual activities and the teaching schedule were removed from each log file, leaving only the records for the individual exercises, lectures and exams. The activities were created by the lecturer so that they filled the whole lesson of 90 min. After cleaning the data, approximately 200,000 records remained for all students^84^.
  During the Quiz activity, there were cases where students answered some questions in a very short time. For the values of the HR to show the effect of the difficulty of the question, etc., a time interval is needed, which we determined to be 6 s. The minimum time interval was set based on the minimum calculated averages of the time needed to answer the questions, the number of words in the question and the possible answers. Students who answered a question in less than 6 s probably chose the answer without reading and understanding the question, and therefore their HR record of the question is not relevant.
3. *Completion of records* input data had to be completed. Since the smart wristbands recorded the HR only when the HR changed or a time window of a maximum of 10 s was reached, the missing records for each second based on the last measured HR had to be completed for processing of further analyses and consistency of records^85^. This was done because the HR of the person did not change and the wristband saves energy this way.
  The record completion methodology is based on the assumption that if the state S1 was measured at time t1, the wristband will record the change of state to state S2 at time t2. This means that the state did not change by time t2 and state S1 was valid. Based on this assumption, we can add records with time ti, where t1 ≤ ti <t2, by the value of state S1. Similarly, we can add state S2 for all records up to time t3 (another state change record from the wristband), when a new state S3 is already acquired, etc.
  For easier handling of time records, the time stamp has been transformed into Unix time. Collections were used to store data in the program, and browsing was secured using an iterator. These values were always supplemented based on the previous record because we assumed that there was no change in HR in the missing time, as explained above. We also set a time window to limit the maximum length of missing records that need to be completed. Since the wristband worn on the student’s wrist created records with a maximum time variance of 10 s (according to the manufacturer), the limit defined by us was set at 60 s, as in practice there were occasionally intervals of a little more than 10 s in the records, which did not mean the end of the session. The pseudocode used is shown in Fig. 3. When completed, the data matrix contained approximately 1,560,000 records for all students together.
4. *Merging input files* Merging data files for each student separately into a single data matrix involved creating a new variable that represented the identifier of the individual student to whom the wristband was assigned. Based on the given identifier, we can combine the data from the wristbands with the data from Moodle and at the same time create new artificial variables that represent information on whether it was an exercise, a lecture, a self-test, or an exam. In this way, it was possible to assign to each record the results from individual self-tests as well as the results from the whole semester and the exam without knowing the name of the student.
5. *Data transformation* involved the creation of variables that were used to examine various factors in combination with the HR of smart wristbands. Such variables included, for example, information about exercise minutes/lecture, activity at a given time, average HR, the difference in HR at a given time compared to average HR, etc. (described in more detail in the next sections of the article).

### Data analysis

HR records may behave slightly differently for different individuals. Our goal is not to explicitly monitor HR levels but rather observe changes in HR levels during various events, activities, etc. However, each person can have different HR levels in a neutral or resting state. If we compare the change in HR for a person who has resting HR (or call it “normal” because we do not have to talk directly about resting HR but rather about the normal HR of the person during the teaching process) of 70 and a person who has 90, then let’s say an HR value of 80 will mean an increase of 10 from the normal value for the first person but it will mean a decrease of 10 compared to the normal value for the second user. It is, therefore, necessary to determine what is normal HR for a given individual, so that we can observe the differences in the measured values of HR compared to the normal HR of the user.

For this purpose, we exported the HR records of each user (since these were records from the teaching process, we are not talking about “resting HR” but rather about normal or “average HR” which we will use as a reference value). Based on these records, we calculated the average HR for each user. The results can be seen in the variable HR_Means in Table 1, where we also indicate the number of records for which HR_Means were calculated and the standard deviation.

**Table 1:** Average HR for individual users.

Student_id	HR_Means	HR_N	HR_Std.Dev.
1	96.298	73,077	12.956
2	73.081	74,318	9.788
3	90.671	64,452	12.342
4	96.139	46,611	9.628
5	88.588	47,068	8.034
7	85.354	78,441	11.164
8	86.982	74,892	11.244
9	89.518	69,980	9.232
10	82.380	51,037	13.186
11	76.398	62,127	7.819
12	80.050	50,236	14.857
13	79.205	37,349	13.382
14	76.641	16,263	11.700
15	77.112	30,171	12.533
16	72.706	20,292	13.628
17	75.024	59,461	10.905
18	86.930	27,653	10.449
19	88.667	61,851	9.671
20	71.463	30,418	15.180
21	76.348	62,749	11.971
22	82.386	53,641	15.372
23	78.478	64,224	8.569
24	73.222	65,923	8.216
All Grps	82.649	1,222,234	13.503

**Figure 4:**
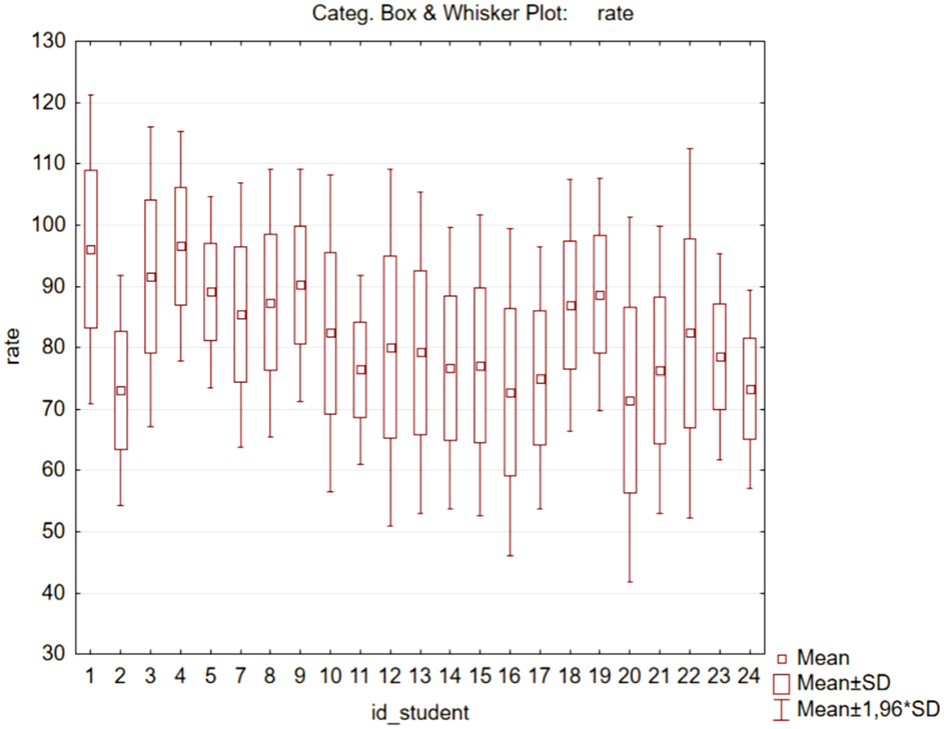
Box and whisker plot for the HR (rate) variable for each user. The graph shows the heart rate values of all respondents involved in the research.

Figure 4 shows that the normal HR of users is quite different and normalization is therefore really needed. We considered the average HRs from Table 1 to be the reference for the given user. This means that, based on these values, we assigned each user a coefficient expressing its reference HR. Further analyses will then be based only on differences from this reference average HR. On this basis, we created new variables in the data matrix, which were calculated as the differences of the currently measured HR from this average HR (HR_AVG). This created the variable HR_Diff (calculated as HR_AVG-rate; i.e. the deviation of the current HR from the average HR in units of beats per minute). This allowed us to compare students’ HRs (in the form of a difference, i.e., the variable HR_Diff) with each other, even though their average HR is different.

The created data matrix (Table 2) was the source of data for further analyses. The raw data matrix, which we processed for further research, contained variables:

- id_student—unique identifier of the student;
- id_session—identifier of the student’s session in the lesson;
- unixtime—the time this record was created;
- HR—heart rate measured for a given student at a given time;
- HR_AVG—average HR of the given student (see Table 1);
- HR_Diff—the difference between the average HR of a student (HR_AVG) and the currently measured HR of the student (HR);
- group—designation of the group to which the student belonged;
- original—an indication of whether the record was measured directly with a wristband or was calculated using the methodology explained in section D. Cleaning and preparation of data, 2. Record cleaning;
- lessonMinute—an indication of how many minutes of the lesson the record belongs to.

**Table 2:** Data matrix (sample).

id_student	id_session	Unixtime	HR	HR_AVG	HR_Diff	Group	Original	LessonMinute
8	136	1,602,138,087	93	86.982	5.637	2	0	51
8	136	1,602,138,086	93	86.982	5.637	2	0	51
8	136	1,602,138,085	93	86.982	5.637	2	0	51
8	136	1,602,138,084	93	86.982	5.637	2	0	51
8	136	1,602,138,083	90	86.982	2.637	2	1	51
8	136	1,602,138,082	90	86.982	2.637	2	0	51
8	136	1,602,138,081	90	86.982	2.637	2	0	51
8	136	1,602,138,080	90	86.982	2.637	2	0	51
8	136	1,602,138,079	90	86.982	2.637	2	0	51
8	136	1,602,138,078	90	86.982	2.637	2	0	51
8	136	1,602,138,077	90	86.982	2.637	2	0	51
8	136	1,602,138,076	89	86.982	1.637	2	1	51
8	136	1,602,138,075	89	86.982	1.637	2	0	51
8	136	1,602,138,074	89	86.982	1.637	2	0	51
8	136	1,602,138,073	89	86.982	1.637	2	0	51
8	136	1,602,138,072	89	86.982	1.637	2	0	51
8	136	1,602,138,071	88	86.982	0.637	2	1	51
8	136	1,602,138,070	88	86.982	0.637	2	0	51
8	136	1,602,138,069	88	86.982	0.637	2	0	51
8	136	1,602,138,068	88	86.982	0.637	2	0	51
8	136	1,602,138,067	88	86.982	0.637	2	0	51
…								

We further modified this as needed to evaluate individual research questions. These adjustments will be described in their evaluation.

Data analysis includes the analysis performed on the obtained, cleaned and prepared data from the wristbands and the log file from the Moodle course. A more detailed data analysis is described in the following section.

Evaluation of results represents partial and final results obtained based on data analysis. The results of the research are described in more detail in the following sections.

## Results

The further analysis aimed to verify which of the types of activities evoke the highest level of arousal. In the context of our research, in which we measure the largest positive change in HR from the average user HR (the highest values of HR_Diff).

Given that we did not use HR directly but the variable HR_Diff, we could use the results of individual users in a common set. Therefore, we took the results of values from HR_Diff and for each student, we calculated the average value of HR_Diff in each implemented activity separately.

Table 3 shows the average measured values of HR_Diff in individual activities. The table contains the variable Activity which is the name of a specific activity in the course, and each of them is also assigned a category. Variables 1–10 contain the average HR_Diff of the student with the given ID during the given activity. Mean_HR_Diff is the average of these values and expresses the average measured HR difference for a given activity. Some cells for a student/activity combination do not contain any value because the student did not participate in the exercise, the record contained incorrect data, or there were insufficient numbers for the given student/activity combination to have sufficient informative value. Based on these values, we subsequently evaluated whether there is a statistically significant difference between the HR_Diff values of different activity categories.

**Table 3:** Average measured HR_Diff for each student and each activity (sample).

lhActivity	Category	Mean_HR_Diff	1	2	3	4	5	7	8	9	10
activity_pl01_02	Activity	0.23	− 3.4	− 6.4	− 0.1	− 1.9	− 0.7	− 0.7	8.6	7.8	− 1.1
activity_pl03_03	Activity	− 1.19	− 17.4	− 4.1	3.3	− 1.4	− 0.6	4.6	2.0	− 5.7	8.6
activity_pl04_03	Activity	− 4.11		− 1.7	− 3.3	− 23.5	1.1	− 5.1	0.9	− 6.4	5.1
activity_pl04_04	Activity	− 3.08		− 9.5	− 5.6	− 5.5	0.9	− 7.0	3.8	− 3.9	2.2
activity_pl04_05	Activity	− 3.22		− 7.4	− 5.1	− 3.8	− 0.6	− 1.5	1.0	− 9.6	1.4
activity_pl05b_04	Activity	− 3.21	− 21.0	4.5	− 12.8		− 1.6	− 0.1	− 2.1	− 3.9	11.2
activity_pl05b_05	Activity	0.40	2.2	4.0	− 14.4		0.7	− 2.9	2.0	− 2.2	13.8
activity_pl06_02	Activity	0.31	3.2	1.0	− 3.6	1.3	− 1.7	− 4.2	− 4.4	5.0	6.3
activity_pl06_04	Activity	− 1.32	− 0.4	2.7	− 5.3	5.2	− 3.8	− 2.2	− 2.7	− 3.9	
activity_pl07_03	Activity	4.80	16.3	8.2	3.0	7.7		− 3.2	9.2	− 8.5	5.7
activity_pl08b_03	Activity	− 1.43	3.8	1.4	− 13.0		− 0.4	− 1.1	4.4	− 5.0	
activity_pl08b_04	Activity	− 2.49	− 5.3	1.7	− 5.7		− 5.4	− 3.3	5.8	− 5.4	
activity_pl09_02	Activity	2.65	2.1	− 2.3	14.8	6.2	8.9	0.0	1.4	3.7	− 10.8
activity_pl09_04	Activity	− 0.90	− 0.1	− 2.8	− 3.1	7.6		− 6.8	− 2.7	6.7	− 6.1
test10_pl08b_01	Test	0.80	0.1	2.2	− 0.5		− 0.9	2.9	1.4	0.4	
test11_pl10b_01	Test	5.59	4.7	9.0	0.5	3.6	− 2.4	12.5	0.2	11.8	10.5
test3_pl01_01	Test	2.77	2.5	− 1.5	5.4	− 4.2	0.3	5.3	10.2	5.2	1.8
test5_pl03_01	Test	1.35	− 19.0	5.5	7.0	7.8	4.6	0.5	5.7	− 1.1	1.2
test6_pl04_01	Test	1.09		− 6.2	− 5.7	− 0.9	3.4	4.1	5.3	− 2.7	11.5

The research problem we are addressing suggests that there is a statistically significant difference between the categories of activities in terms of the arousal they evoke in students. We, therefore, set out to verify the null hypothesis H0.

### H0

There is no statistically significant difference between the activity categories in the Mean_HR_Diff variable.

To this end, the Mean_HR_Diff values (Table 3) must be compared across activity types: Activity, Test, Lectures, Presentation, Exam, and Other. Given the ordinal nature and non-normal distribution of our data, and considering the comparison spans multiple groups, the Kruskal–Wallis ANOVA test is deemed appropriate. The data meets the essential assumptions for this test: samples across categories are independent, the data is ordinal, and the distribution shapes across groups are approximately similar. As a non-parametric test, Kruskal–Wallis operates on rank orders rather than raw data values, rendering it suitable for datasets that don’t adhere to parametric test assumptions. This test can be perceived as an extension of the Mann–Whitney U test, tailored for more than two independent groups.

The test criterion in Kruskal–Wallis ANOVA is based on ranking. In this method, it is first necessary to rank the data points irrespective of the group they belong to. Subsequently, the original values are replaced with their respective ranks, and a sum of ranks (Sum from Ranks) is computed for each group. The test statistic is then derived using a specific formula^69^:

1 $$H=\frac{12}{N\left(N+1\right)}\sum \limits_{i=1}^{C}\frac{{R}_{i}^{2}}{{n}_{i}}-3\left(N+1\right),$$

where *C* is the number of classes (groups), *n*_*i*_ is the number of observations in the i-th class, N is the number of observations in all classes, *R*_*i*_ is the sum of the orders in the i-th class.

Detailed results are given in Table 4.

**Table 4:** Kruskal–Wallis ANOVA and descriptive statistics for activity categories concerning the variable HR_Diff.

	Valid N	Sum of ranks	Mean rank	Means	Std. Dev.
Activity	19	404	21.263	0.308	2.208
Test	11	419	38.091	3.543	2.129
Other	1	17	17	− 0.388	–
Lecture	6	33	5.5	− 3.793	2.654
Presentation	10	273	27.3	1.478	2.54
Exam	4	180	45	5.045	2.263

A Kruskal–Wallis ANOVA test showed that there was a statistically significant difference in HR_Diff values between the activity categories, χ^2^(5, N = 51) = 27.592, p < 0.001. Thus, the null hypothesis was rejected, indicating variations in the level of arousal across different types of activities.

Based on the result, we can interpret Table 4 in such a way that the students were most aroused during the Exam and Test (self-test) and the least during the Lecture. As the Exam represented the final evaluation of the subject, it evoked the highest level of arousal in the students. The test was an activity that students carried out at the beginning of the teaching process. For students belonging to the experimental group, the results from the Test were counted in the final evaluation (grades), and this was the reason why they showed a higher level of arousal. The lecture was part of the teaching process, where the teacher taught the theoretical part of the subject to students, without active student interaction, and therefore students showed a lower level of arousal.

**Figure 5:**
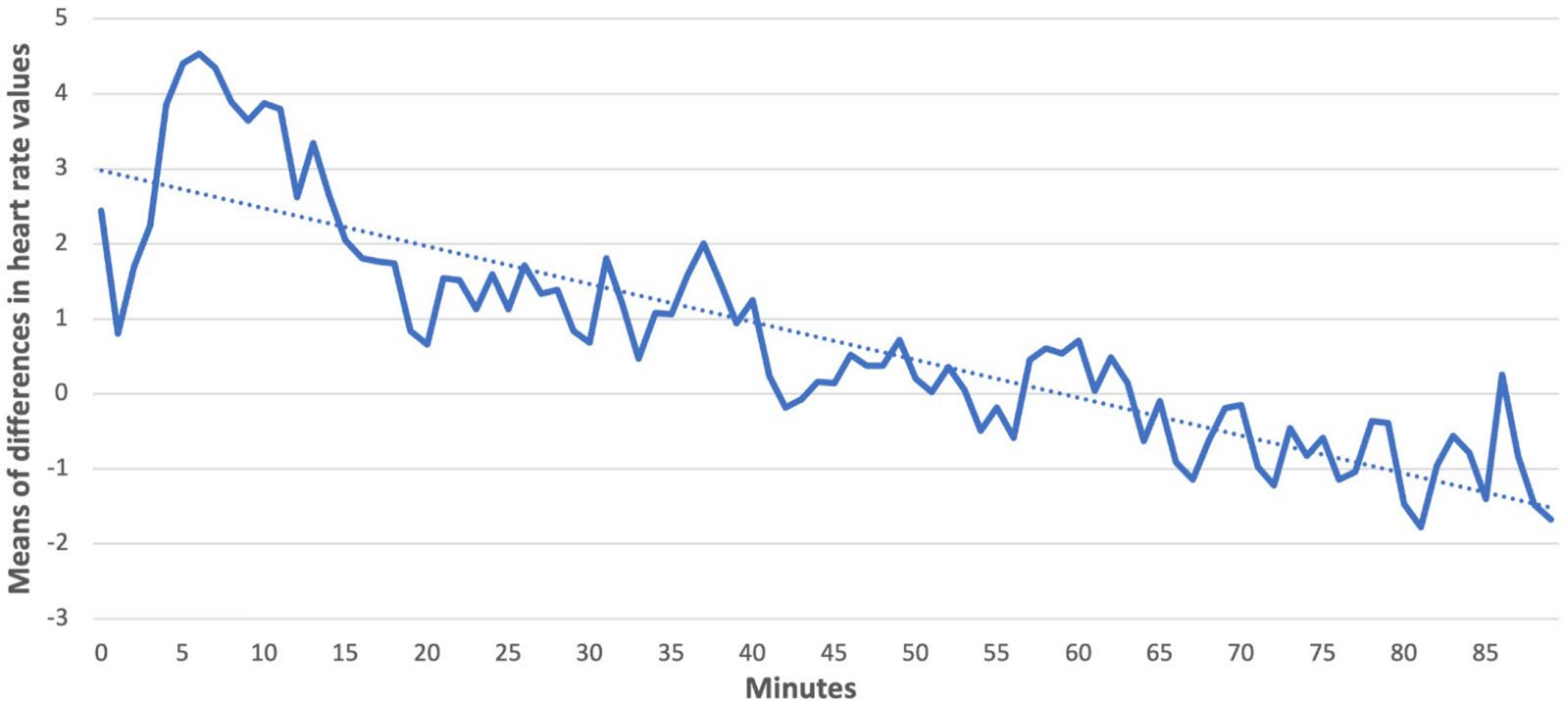
Averages of student HR during the exercise. Graph showing the decreasing value of students’ heart rate during the teaching process.

Subsequently, we compared the students’ behavior during the exercises. Each exercise began with a self-test and continued with a presentation with an introduction to the issues and assignments that students addressed during the exercise. At the beginning of the exercise when writing the self-test, the students were more aroused and in the further course of the exercise, they were calmer, as evidenced by the difference in HR values during the teaching process, shown in Fig. 5. We used the average of the measured heart rate data^84^.

We performed a more detailed analysis during the self-test activity where we found out the behavior of students from the perspective of the physiological function of the HR.

**Figure 6:**
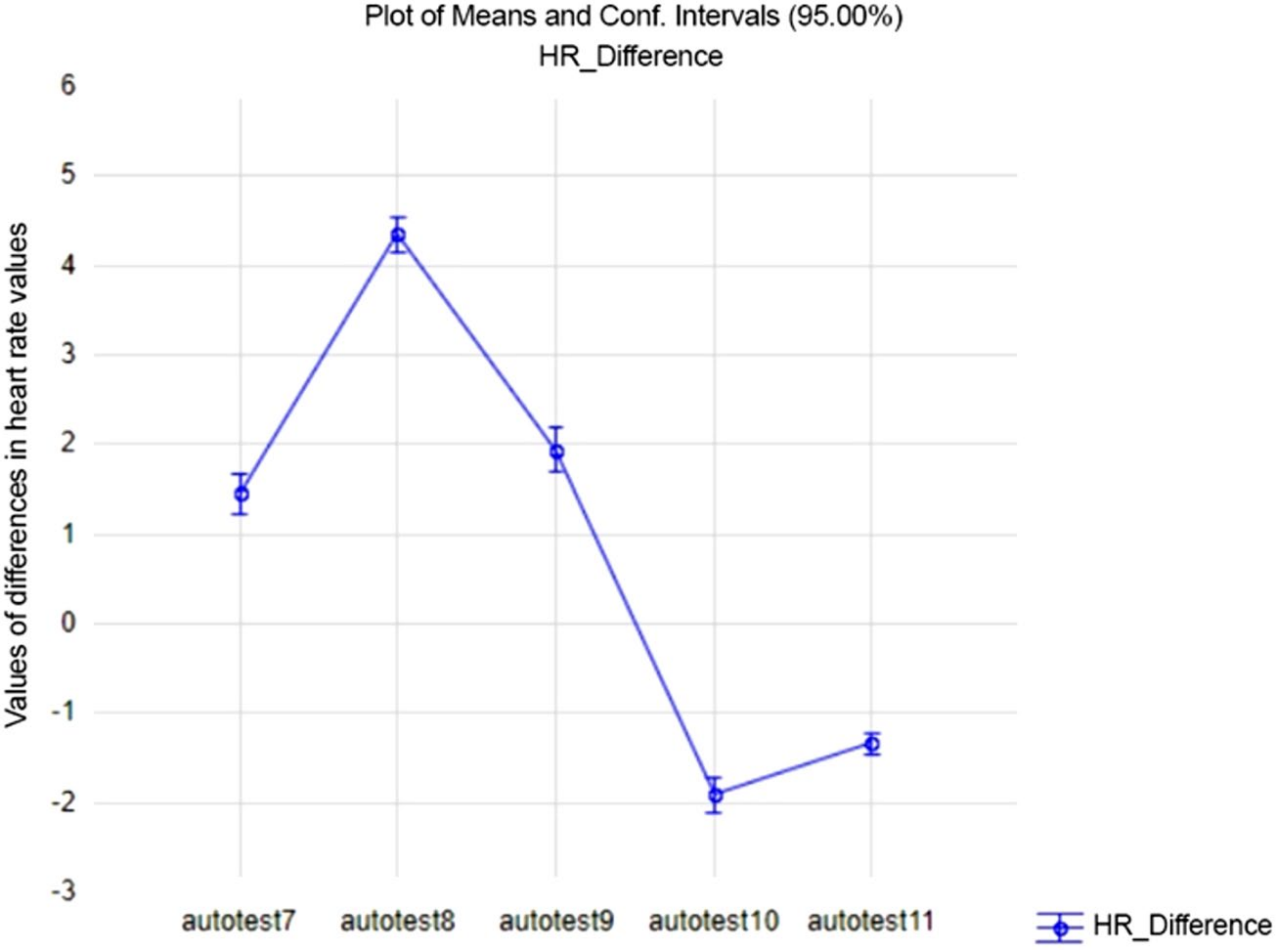
Arousal differences of students according to self-tests. The values in the graph show the level of HR_Difference based on the self-tests taken during the semester.

From Fig. 6 we can see that on average the students were most aroused in the self-test 8 and calmer in the self-test 10.

**Figure 7:**
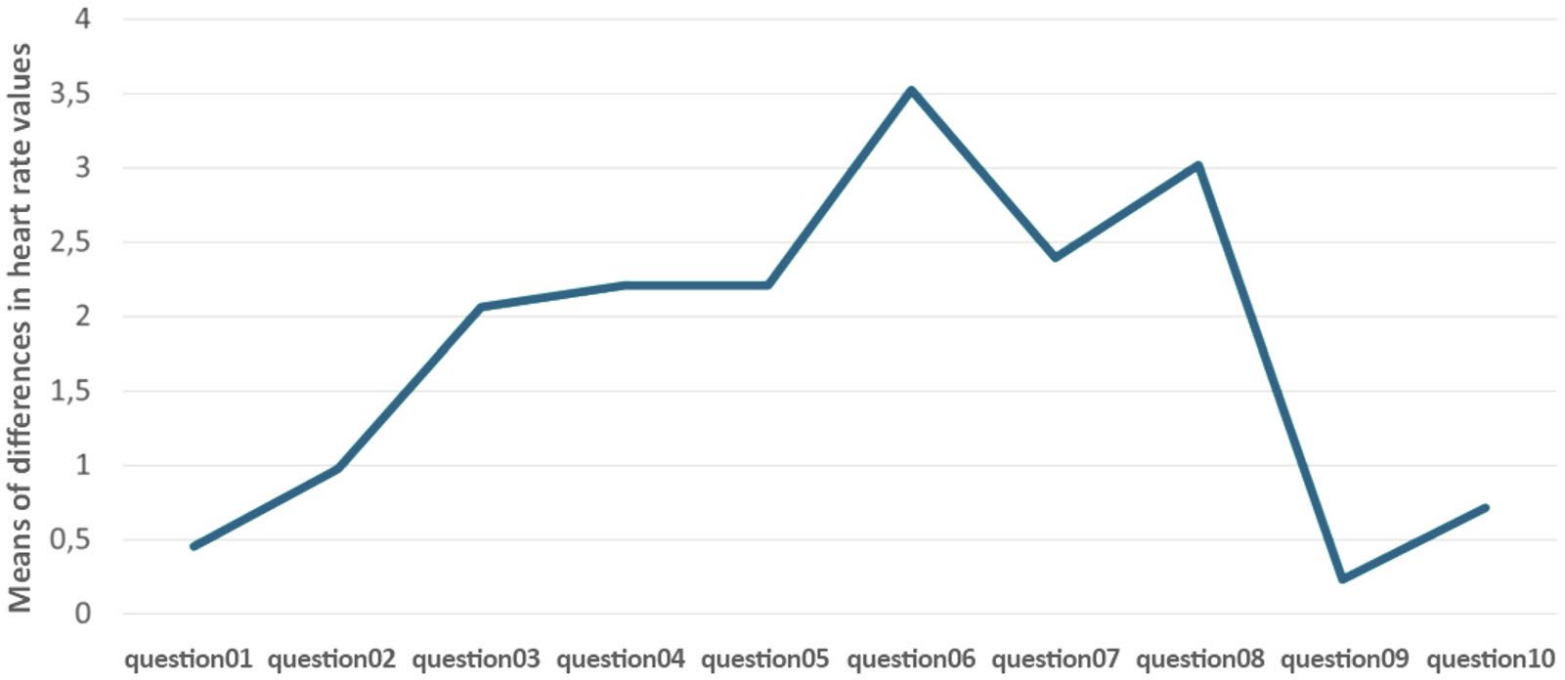
The behavior of students during the writing of self-tests based on heart rate values.

During the self-test activity, we also compared the testing process itself. The self-tests were created sequentially, and it was not possible to move freely within the test. From the perspective of the physiological function of the HR, the course of self-tests had an increasing tendency, and with each subsequent question, an increase in the level of arousal was noted in the students, shown in Fig. 7.

The self-test included three types of questions (selection of one correct answer, selection of several correct answers, writing the correct answer).

Since the subject on which we carried out the research belonged to the group of compulsory subjects, all students (divided into two groups) had to take this subject. The groups differed from each other, as we mentioned above, in the scoring system. For students who belonged to the experimental group, the points from the self-tests were counted in the final grade. Based on the division into two groups (control and experimental), we performed further analyses, compared two different groups, focusing on the self-test activity, preparing students for the activity and the overall activity of students in the course.

In the first step, we compared the behavior of groups of students during the self-test activity. We found that students belonging to the experimental group, to whom the grades from the self-test were counted into the final grade (information about which they were acquainted) showed 25% higher arousal during writing the self-test compared to students from the control group who wrote the self-test as repetition.

In addition to the results in terms of the physiological HR function, we also compared the groups based on the points obtained from self-tests. The obtained averages of the results from the self-tests were almost identical between the groups, the students from the experimental group were 0.5% more successful, which does not represent a statistically significant difference.

We also made the comparison from the perspective of group preparation for self-tests. Students from the experimental group prepared more for self-tests according to the number of clicks recorded in the course, which we can also see in Table 5.

**Table 5:** The number of clicks in the course in the time interval between the lecture and the exercise.

Autotest	Control group	Experimental group
Autotest5	362	411
Autotest6	110	359
Autotest7	163	220
Autotest8	283	388
Autotest9	233	351
Autotest10	322	229

We performed an analysis of the test results for individual groups. The resulting grades between the groups were almost identical on average, the students from the control group were on average 3% more successful (Table 6), which, similarly to the results from the self-tests, does not represent a statistically significant difference.

**Table 6:** Exam results between groups.

Groups	Control group	Experimental group
Score in %	75.51%	72.4%

The obtained results directly helped us to confirm the statement that based on the physiological function of the HR, it is possible to identify changes in the levels of arousal of students. We also found that students’ behavior influenced their HR during each activity.

## Evaluation and discussion

Sensory networks are a powerful utility that allows the creation of an overall complex system. It can automatically fulfil the specified purposes, in our case the collection of data of physiological functions in favor of creating recommendations for the creation of an adaptable teaching process.

Similar solutions and research results were also achieved by the authors Carroll et al., who dealt with the automatic detection of student engagement using machine learning and wearable sensors. They describe the development and testing of a measurement and classification technique that uses non-invasive physiological and behavioral monitoring technology to directly assess engagement in classroom, simulation and live training environments^75^.

As part of the research, students were divided into two groups, experimental and control. The main goal of the division of groups was to compare their behavior in terms of the physiological HR function. Based on analyses of the average differences in HR values, we found that students belonging to the experimental group were more aroused when writing self-tests compared to students from the control group. Students who were in the experimental groups were more aroused because their self-test results were included in the final evaluation compared to the control group, to whom the self-test results were not included in the final evaluation. They were informed of these facts in advance. Although the students from the control group were repeatedly informed that the points from the self-tests are counted toward the final grade, they achieved on average 3% better results from the self-tests. Even though the students from the experimental group were acquainted with the inclusion of the results from the self-tests into the final grade (evaluation), they did not show a greater effort (activity) to fill out the self-test better. Although this was reflected in their HR, the students in the experimental group were more aroused but they did not achieve a significant difference in the points obtained compared to the students in the control group.

In addition to self-tests, the average differences in HR values for other activities were compared, e.g., presentation or activity where students worked independently. From the results of the analyses, we found out that the students were more aroused when presenting to the teacher during the exercises than during the independent activity. The higher value of arousal may have been due to students being asked to answer a question they may not understand. This statement was also confirmed by the students in the output questionnaire.

We found out that the identified changes in the levels of arousal can be an identifier of activities, during which students are more aroused and this information can be used to adjust activities to make students calmer and more focused in the future.

Based on the results of the Kruskal–Wallis ANOVA, which are shown in Table 4, we found that the students were most aroused during the Exam and Test activities and the least during the Lecture. The results confirmed the rejection of the null hypothesis, which as an assumption claimed that there is no statistically significant difference between the differences in HR values (HR_Diff) during different activities in the teaching process. Rejection of this hypothesis means that what activity students deal with during the lesson affects their HR values. The results also indicate that these HR_Diff values behaved as expected. Thus, during the lesson, where students are rather passive observers and there is no requirement for the significant activity, HR_Diff values were at low levels. In contrast, for the Exam or Test activities, HR_Diff values were above average, indicating a significant increase in HR during the test or self-test.

These results and conclusions are intuitively logical, but the results of this experiment also scientifically confirm this assumption. This means that although the measurement of the HR speaks of only one specific physiological activity of the student’s body, it can be used to verify the physiological activity of students in various types of activities in another research. This data alone cannot identify, for example, an emotional state, but as additional data, it can be very useful, as the results of the experiment suggest.

A similar experiment was performed by the authors^76^, who noted the possibility of using wearable sensing devices to automatically capture students’ learning experiences. Provides timely feedback to both students and teachers. It thus enables teachers to receive early warnings of low mental effort, disengagement and disinterest, and enables students and teachers to take corrective action very early.

In research, we also found that the physiological function of HR can be used as an identifier to identify changes in students’ arousal levels. Smart wristbands can also be used as a tool to measure the physiological function of HR.

## Conclusions

The research aimed to show that physiological functions can be used as an identifier of changes in students’ arousal level during the teaching process. The aim was also to show that a change in physiological state (HR) affects a change in the level of arousal of the student, which in the case of a positive change could also lead to stressful situations.

Through the obtained data, we managed to successfully carry out research similar to others, which are described in the “Related work” section. The final result of the research created the preconditions for the creation of a software utility designed to optimize the teaching process.

During the research, students had wristbands worn during each teaching process, so we could observe and compare the values obtained with the activities. We assumed that students would be more aroused during the interaction with self-tests and the Exam, as in the experimental group, the points from the self-tests were also counted in the final grade, and this was confirmed based on measured data.

The results of the research showed that a certain level of arousal during the writing of self-tests and exams was present in both groups of students, “Means” column shown in Table 4. However, the level of arousal was 25% higher for students from the experimental group, compared to students from control group. We found that a higher level of arousal did not provide a higher degree of success of the final exam (control group 75.5% and experimental group 72.4%). Therefore, no activities must be created during the teaching process in which students can signify a high level of arousal.

Based on the findings, it follows that it is more beneficial for students to carry out self-tests as a repetition of teaching materials obtained in lectures without counting the points obtained in the final grade.

We found that the way we conducted research can be used to optimize educational processes with an emphasis on students’ success in the teaching process.

In the future, an effort is going to be made to create a system that, based on acquired physiological functions in real-time to identify changes in the degree of arousal of students and would serve as a software utility for the teacher, for real-time visualization of how students behave in a particular activity and whether to edit an activity^86^.

A comprehensive tool, which can be created based on the research data, can be applied in addition to the teaching process in other industries, where it is possible to use an intelligent wristband to measure the physiological function of the HR. To specify and subsequently classify emotional states, it is necessary to add additional sensors that measure physiological functions such as GSR, Face recognition, Thermal-camera, EEG, etc.

Despite the pandemic situation (COVID-19), we were able to obtain data from physiological function (HR), evaluate this data and in the future, it is going to be possible to solve similar situations and thus remotely (in distance learning) adapt teaching style, adapt teaching materials and optimize teaching process.

## Data Availability

The data will be available after the acceptance of the paper on a public GitHub repository. You can access the data in the draft version: https://github.com/ChrisFodor333/WristBandsDataset/blob/main/dataset_wristbands.zip.
